# Human Dominant Disease Genes Are Enriched in Paralogs Originating from Whole Genome Duplication

**DOI:** 10.1371/journal.pcbi.1003754

**Published:** 2014-07-31

**Authors:** Param Priya Singh, Séverine Affeldt, Giulia Malaguti, Hervé Isambert

**Affiliations:** CNRS-UMR168, UPMC, Institut Curie, Research Center, Paris, France; Cornell University, United States of America

*PLOS Computational Biology* recently published an article by Chen, Zhao, van Noort, and Bork [1] reporting that, in contrast to duplicated nondisease genes, human monogenic disease (MD) genes are (1) enriched in duplicates (in agreement with earlier reports [2]–[5]) and (2) more functionally similar to their closest paralogs based on sequence conservation and expression profile similarity. Chen et al. then proposed that human MD genes frequently have functionally redundant paralogs that can mask the phenotypic effects of deleterious mutations.

We would like to point out here two lines of evidence that appear more relevant to the explanation of this surprising enrichment of human disease genes in duplicates. The first line of evidence indicates that human gene duplicates should be distinguished depending on whether they originate from small-scale duplication (SSD) or from the two rounds of whole genome duplication (WGD) that occurred in early vertebrates some 500 million years ago. In fact, as shown quantitatively below using Chen et al.'s dataset, human MD genes are actually depleted, not enriched, in SSD duplicates, whereas they are clearly enriched in WGD duplicates when compared to nondisease genes. This opposite retention pattern cannot be explained by a selection mechanism independent of the SSD or WGD origin of MD gene duplicates. The second line of evidence concerns the mode of inheritance of human MDs, which provides a more stringent criterion than sequence conservation or coexpression profile to assess the likelihood of functional compensation by paralogs of MD genes. In particular, the recessiveness of a human disease is expected to be a prerequisite for functional compensation by a paralog gene. Indeed, autosomal dominant MDs are unlikely to experience significant functional compensation from a different locus, since even a perfectly functional allele is unable to mask the deleterious phenotypic effects of a dominant allelic mutant on the same heterozygote locus.

We first address the difference between SSD duplicates and WGD duplicates, also called “ohnologs” after Susumu Ohno's early “2R hypothesis” [6], which has now been firmly established [7]. The importance of distinguishing between SSD and WGD duplicates in the human genome has already been reported in a number of papers [2]–[4], [8], including our own [5], [9]. As shown in Figure 1A, human genes tend to partition into three main gene categories with respect to duplicates: those with WGD but no SSD duplicates (about 28%), those with SSD but no WGD duplicates (about 41%), and singletons without WGD or SSD duplicates (about 24%), while human genes with both WGD and SSD duplicates are relatively rare (about 7%). Gene families enriched either in WGD or SSD duplicates also correspond to distinct functional classes [2], [8], with WGD genes frequently involved in signaling, regulation, and development, whereas SSD genes are typically implicated in different functions such as antigen processing, immune response, and metabolism.

**Figure 1:**
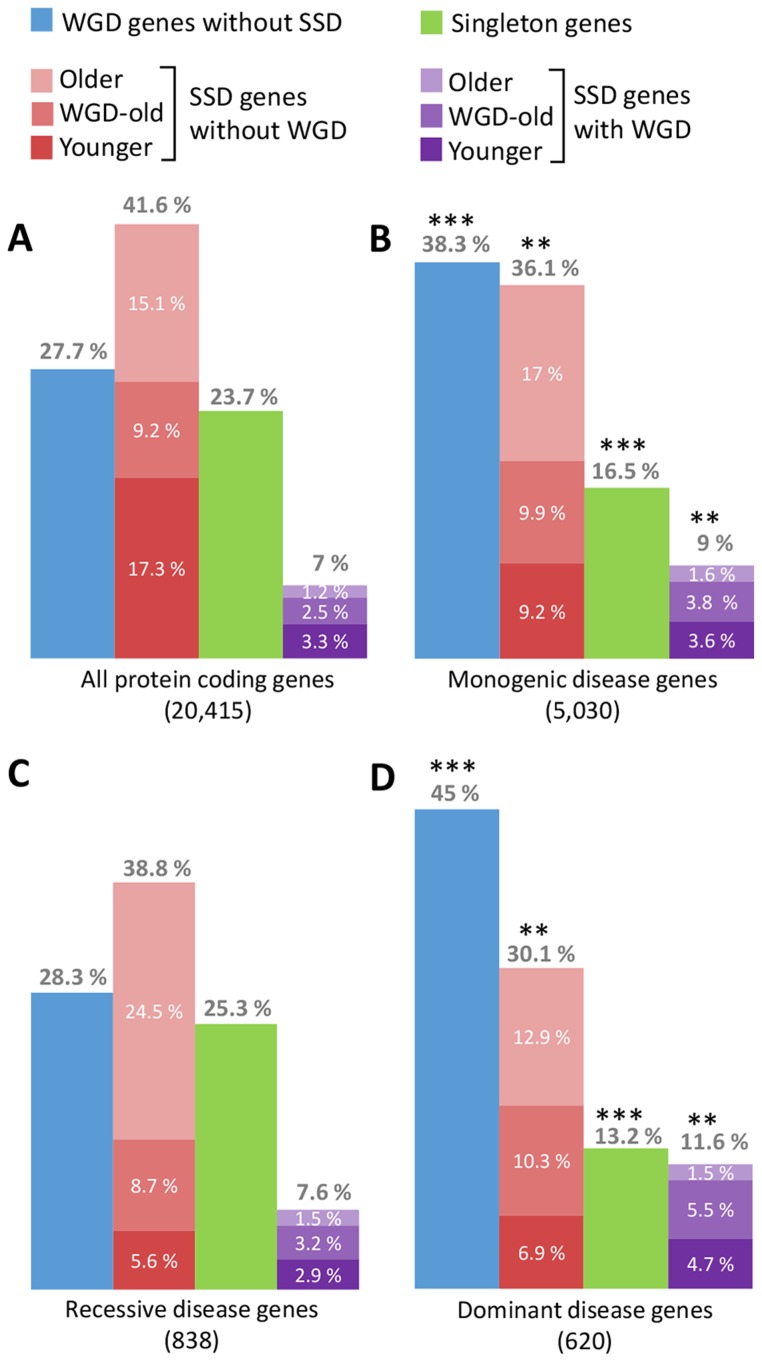
Distributions of WGD, SSD, and singletons in (A) the whole human genome, (B) monogenic disease (MD) genes [1], (C) recessive MD genes, and (D) dominant MD genes. (***) corresponds to highly significant deviations (p<10^−6^, FE test) and (**) to significant deviations (p<10^−3^, FE test) from the references in (A). Note that recessive MD genes (C) do not show any significant deviations in WGD, SSD, or singleton contents (p>0.3, FE test), although taking into account the age of SSD duplicates reveals a relative lack of recent SSD genes in MD genes (see text).

In addition, human disease genes have been shown to be significantly enriched in WGD duplicates, while they are rather depleted in SSD duplicates [2], [5], [8], [9]. This could not be seen with Chen et al.'s dataset, which lumps together all gene duplicates irrespective of their WGD or SSD origin. In fact, using the same monogenic disease (MD) dataset, we could readily extend these earlier results, as depicted in Figure 1B. MD genes are significantly enriched in ohnologs, 38.3% versus 27.7% (p = 1.58×10^−25^; Fisher's Exact [FE] test), while showing at the same time a significant depletion in both singletons, 16.5% versus 23.7% (p = 7.67×10^−20^; FE test), and SSD, 36.1% versus 41.6% (p = 2.75×10^−6^; FE test). MD genes are more specifically depleted in recent SSD, 9.2% versus 17.3% (p = 4.1×10^−50^; FE test), while WGD-old and older SSD of MD genes are not significantly biased, i.e., 9.9% versus 9.2% (p = 0.12; FE test) and 17% versus 15.5% (p = 0.001; FE test), respectively (see below). These results demonstrate that, although MD genes retain significantly more duplicates than singletons (Figure 1B), these duplicates are primarily enriched in ohnologs and not SSD copies, as compared to the relative WGD and SSD content of the entire human genome (Figure 1A, Dataset S1).

To explain the global enrichment in MD gene duplicates, Chen et al. noticed that coexpressions between MDs and their closest paralogs are in general higher than that of nondisease genes (p = 0.00298, Figure 2B in [1]), which they interpret as evidence that “functional compensation by duplication of genes masks the phenotypic effects of deleterious mutations and reduces the probability of purging the defective genes from the human population.” In particular, the retention of MD gene duplicates should be favored by the higher functional redundancy of recent, less-diverged duplicates. However, investigating the age of SSD duplicates from MD genes suggests rather the opposite, as MD genes tend to have fewer recent SSD than old SSD duplicates, as compared to nondisease (ND) genes (Figures 1A and B). In particular, focusing on genes with SSD but no ohnolog, we found that 9.2% [respectively 17%] of MD genes have SSD that are more recent [respectively ancient] than the two rounds of whole-genome duplication, while the overall genome exhibits 17.3% [respectively 15.1%] instead (p = 4.5×10^−34^; FE test). This suggests that the functional compensation, which can occur between functionally redundant duplicates, leads to a depletion (not an enrichment) of MD genes with recent SSD, in agreement with an earlier report [10].

**Figure 2:**
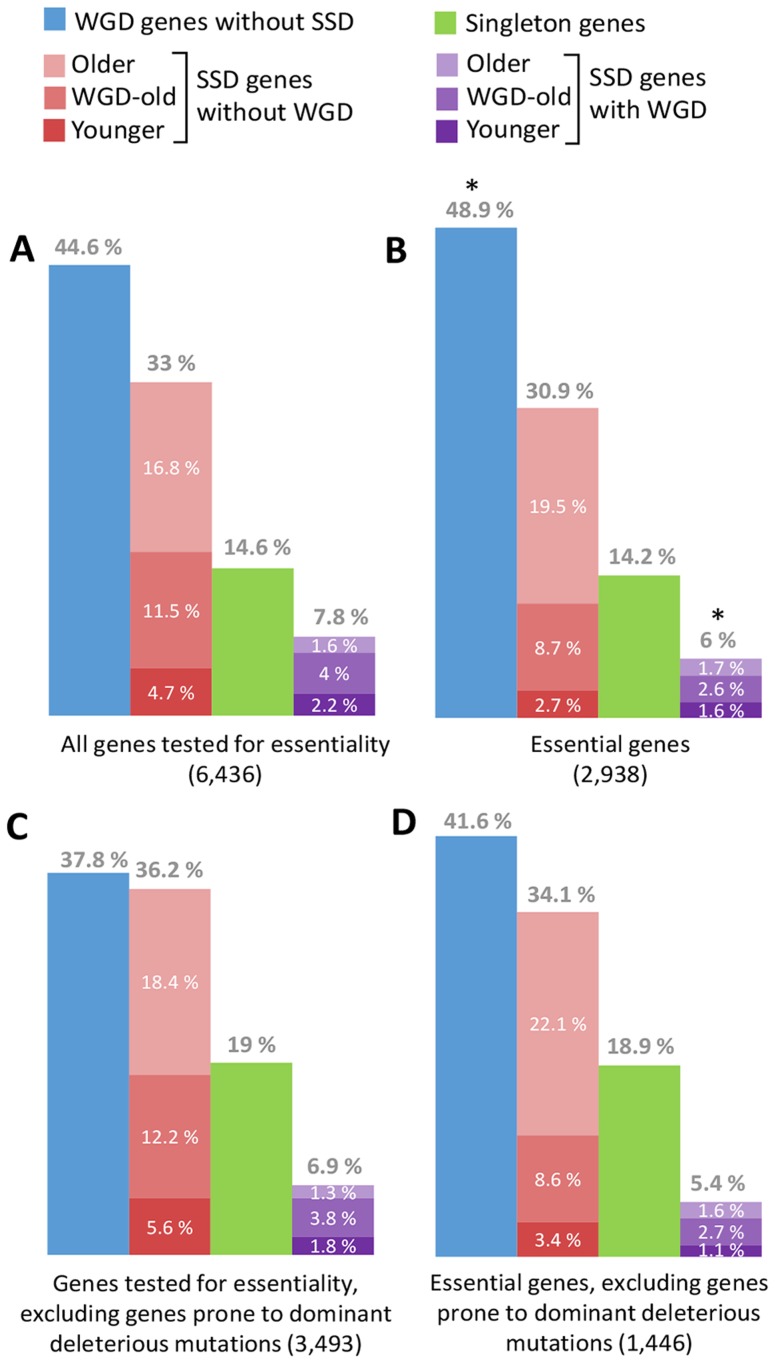
Distributions of WGD, SSD, and singletons for human orthologs of mouse genes (A) tested for essentiality in mouse [13], (B) found to be essential in mouse, and (C and D) after removing dominant disease genes, oncogenes, and genes with dominant negative mutations or autoinhibitory folds [5]. (*) corresponds to small deviations (10^−3^<p<0.05, FE test) from the references in (A). Note that human orthologs of essential genes in mouse do not show any significant deviations in WGD, SSD, or singleton contents (p>0.05, FE test) once dominant disease genes, oncogenes, and genes with dominant negative mutations or autoinhibitory folds have been removed. Yet, taking into account the age of SSD duplicates reveals a relative lack of recent SSD genes in essential genes (see text).

In addition, we note that, while recent gene duplicates might be able to mask the phenotypic effect of recessive (e.g., loss-of-function) mutations, dominant (e.g., gain-of-function or dominant negative) mutations should typically lead to deleterious phenotypic effects regardless of the presence of any functionally redundant paralog at a different locus on the human genome.

In order to assess the extent of possible functional compensation on the retention of MD gene duplicates, we have thus investigated the mode of inheritance of human MDs. To this end, we retrieved the available information on the dominance and recessiveness of MDs from Online Mendelian Inheritance in Man (OMIM) [11] and Blekhman et al. [12]. Manual curation yielded 620 autosomal dominant and 838 autosomal recessive MD genes after excluding sex-linked genes and MD genes documented as both dominant and recessive (Dataset S1).

Using Chen et al.'s dataset and analysis, we then found that autosomal recessive MD gene duplicates (with possible functional compensation) do not exhibit significantly more correlated expression profiles than ND genes (p = 0.426, Wilcoxon Rank Sum Test, as compared to p = 0.00298 for all MD genes in Figure 2B in [1]), whereas autosomal dominant MD gene duplicates (with unlikely functional compensation) in fact exhibit significant expression profile correlations (p = 0.00028).

Moreover, looking for duplication biases of recessive versus dominant MDs confirmed that recessive MDs, which could in principle provide functional compensation, have not retained significantly more duplicates. Indeed, Figure 1C shows that recessive MDs do not present any biased retention of ohnologs, 28.3% versus 27.7% (p = 0.79; FE test); SSD duplicates, 38.8% versus 41.6% (p = 0.31; FE test); or singletons, 25.3% versus 23.7% (p = 0.42; FE test), as compared to their respective prevalence in the entire human genome (Figure 1A). These observations clearly show that the maintenance of recessive MD genes is largely independent of their WGD, SSD, or singleton status, suggesting limited effects of functional compensation by paralogs on the retention of gene duplicates associated to recessive MDs in human. By contrast, we observed (Figure 1D) that dominant MDs exhibit a strong enrichment in ohnologs, 45% versus 27.7% (p = 1.8×10^−10^; FE test), with concomitant depletions in both SSD, 30.1% versus 41.6% (p = 0.0001; FE test), and singletons, 13.2% versus 23.7% (p = 1.59×10^−7^; FE test). The same trend is observed for haploinsufficient and dominant negative genes [5]. This is unlikely to result from a functional compensation by paralogs because of the molecular genetics of dominance, as discussed above.

Finally, we investigated the enrichment in WGD and SSD duplicates of essential genes for which functional compensation could in principle be advantageous owing to the lethality of their double mutants. However, we found that human orthologs of mouse genes, reported as being “essential” genes from large-scale null mutant studies in mouse [13], are only slightly enriched in ohnologs, 48.9% versus 44.6% (p = 0.02, FE test), and hardly depleted in SSD, 30.9% versus 33% (p = 0.14, FE test), in which 44.6% and 33% are, respectively, the global proportions of ohnologs and SSD among the 6,436 genes tested for null mutation in mouse (Figures 2A and 2B). In fact, these small deviations, consistent with earlier findings [14], even become nonsignificant once genes with dominant allelic mutants are removed from the list of 6,436 genes tested for essentiality in mouse (Figures 2C and 2D), i.e., 41.6% versus 37.8% for ohnologs (p = 0.1, FE test) and 34.1% versus 36.2% for SSD (p = 0.34, FE test), in which 37.8% and 36.2% are, respectively, the global proportions of ohnologs and SSD among the 3,493 genes tested for null mutation in mouse after removing dominant disease genes, oncogenes, and genes with dominant negative mutations or autoinhibitory folds (Figures 2C and 2D) [5]. Hence, we could not find any significant enrichment in duplicates in support of possible functional compensation for essential genes, in broad agreement with early reports [15], [16]. Moreover, taking into account the age distribution of SSD duplicates (including corrections for the visible age bias of genes tested for essentiality in mouse [Figures 1A and 2A]) [17], [18], we actually found a relative lack of recent SSD of essential genes (Figure 2B and 2D) as observed for MD genes (Figure 1B–1D). This suggests evidence against functional redundancy in vertebrate essential genes, in agreement with an earlier report [17] and similar observations in yeast [19] and nematodes [20].

So, what could be the evolutionary mechanism behind the enhanced retention of WGD duplicates and relative depletion of SSD duplicates and singletons associated to MDs in humans (Figure 1B)? In other works [5], [9], we proposed a population genetics model based on the observation that a major difference between SSD and WGD scenarios concerns the timing of fixation of gene duplicates. It is well-known that the SSD scenario starts with a gene duplication in the genome of a single individual, which subsequently needs to spread through the entire population to reach fixation. By contrast, the WGD scenario entails an initial fixation of duplicated gene pairs in the genome of all individuals in the small population, arising through WGD. This is because WGD typically induces a speciation event due to the ploidy incompatibility of the post-WGD individuals with the rest of the pre-WGD population. This population genetics model [9] for the fixation of SSD versus WGD duplicates then predicts that the enhanced retention of “dangerous” ohnologs prone to dominant deleterious mutations (as depicted in Figure 1D) is a direct consequence of purifying selection in post-WGD population, as most surviving individuals retain (nondeleterious) functional copies of their ohnologs that are prone to dominant deleterious mutations. By contrast, ohnologs prone to recessive deleterious mutations are more readily eliminated through loss-of-function mutations and are not expected to exhibit significant ohnolog retention bias (in agreement with Figure 1C). As for SSD duplicates, they are expected to be retained either from adaptive selection in large populations (N>10^5^) or from purifying selection in small populations (N<10^4^), in which SSD duplicates typically reach fixation by drift before their mutations actually occur, hence resembling the WGD scenario with an initial fixation of ohnologs through speciation in that case. This leads in principle to a complex retention pattern of SSD duplicates across evolutionary ages, in particular around WGD-induced population bottlenecks. Yet, overall it appears that genes with recent SSD duplicates are less likely to be MD genes than genes with WGD-old or older SSD duplicates (Figure 1).

All in all, we found that MD genes have preferentially retained WGD rather than SSD duplicates, as compared to nondisease genes. Yet, only dominant MD genes exhibit a clear enrichment in WGD duplicates, while the retention of duplicates of recessive MDs or essential genes, which might in principle experience functional compensation from paralogs, is in fact largely independent of their WGD, SSD, or singleton status. These results cannot be explained by the functional compensation hypothesis proposed in Chen et al. [1]. They are, however, consistent with a population genetics model taking into account the initial fixation of ohnologs through WGD-induced speciation and the ensuing purifying selection in post-WGD populations [5], [9].

## Materials and Methods

We obtained 20,415 protein coding genes in the human genome from Ensembl version 70 (Dataset S1). Ohnologs (7,075 genes) were obtained from [4], and SSDs (9,916 genes) were obtained by running an all-against-all BLASTp using the human proteins (see [5] for details). The genes which could not be classified as ohnologs or SSDs were taken to be the singleton genes (4,846 genes). The duplication timing of SSD genes was obtained from Ensembl compara [21] using BioMart.

MD genes were taken from Chen et al. [1]. We could map 5,030 of 5,134 MD genes on our dataset using BioMart. Inheritance status of the MD genes were obtained either from the inheritance section from OMIM entries [11] or from Blekhman et al. [12]. After careful manual curation, we could obtain 1,458 MD genes in which the inheritance pattern was unambiguously described as either autosomal dominant (620 genes) or autosomal recessive (838 genes).

Expression profile correlation between autosomal recessive genes and ND genes, and autosomal dominant genes and ND genes was performed using the R scripts provided by Chen et al. [1].

We obtained 6,436 mouse genes tested for null/knock-out mutations from the Mouse Genome Database (MGD) [13], as described in [5]. 2,938 of these 6,436 genes had lethal or infertile phenotypes and were classified as “essential.” Human one-to-one orthologs of these genes were obtained using Ensembl BioMart. We also investigated the enrichment of essential genes in duplicates after removing dominant disease genes. To this end we considered multiple classes of genes susceptible to dominant mutations, including 620 dominant disease genes (from this report), 5,996 oncogenes [22], 566 dominant negative genes, and 461 genes having autoinhibitory folds [5].

## Supporting Information

Dataset S1 **Dataset used for the analysis.** Gene IDs and symbols are from Ensembl v70. Values in the columns correspond to one of the following descriptions: N = no; Y = yes; Onc = oncogene; TS = tumor suppressor; O = others; AD = autosomal dominant; AR = autosomal recessive; XL = X-linked; and YL = Y-linked.
(XLSX)
